# Two cases of primary solitary fibrous tumor in the pelvis resected using laparoscopic surgery

**DOI:** 10.1016/j.ijscr.2020.04.079

**Published:** 2020-05-14

**Authors:** Yuki Matsui, Madoka Hamada, Fusao Sumiyama, Toshinori Kobayashi, Yuki Matsumi, Hisanori Miki, Mitsuaki Ishida, Hiroaki Kurokawa, Mitsugu Sekimoto, Yoko Sekita-Hatakeyama, Kinta Hatakeyama, Chiho Ohbayashi

**Affiliations:** a Division of Gastrointestinal Surgery, Kansai Medical University Hospital, Hirakata, Osaka, Japan; b Department of Pathology and Laboratory Medicine, Kansai Medical University Hospital, Hirakata, Osaka, Japan; c Department of Radiology, Kansai Medical University Hospital, Hirakata, Osaka, Japan; d Department of Diagnostic Pathology, Nara Medical University, Kashihara, Nara, Japan

**Keywords:** Solitary fibrous tumor, Pelvic tumor, STAT6, Fusion gene, Laparoscopic surgery

## Abstract

**Background:**

We report two resected cases of solitary fibrous tumors (SFT) that were accidentally found in the pelvic cavity.

**Case presentation:**

Case 1 was a 54-year-old male. A colonoscopy for the examination of intestinal polyps revealed an extramural tumor in the right anterior wall of the low rectum. A preoperative MRI showed a well-demarcated T1 low and T2 mixed intensity extramural tumor (53 × 36 mm) located right lateral to the low rectum and behind the seminal vesicle. Laparoscopic surgery was successful for tumor extirpation. Immunohistochemical examination of the specimen revealed STAT6 (+) and CD34 (+) cells, a Ki67 positivity of 7–8%, a mitotic index of 4–5/50 HPF, and a diagnosis of SFT. There was no recurrence 29 months after surgery. Using RT-PCR and sequencing, we detected the NAB2-STAT6 fusion gene but the locus of genomic inversion was not detected. Case 2 was a 43-year-old male that received conservative treatment for appendicitis. A CT scan accidentally revealed a tumor of 40 mm of length in the left obturator area. A MRI revealed a well-demarcated T1 and T2 high intensity tumor. The patient underwent surgical biopsy. Immunohistochemical examination of the biopsy revealed STAT6 (+) and CD34 (+) cells, Ki67 positive cells < 1%, and a diagnosis of SFT. We could not detect the NAB2-STAT6 fusion gene in the extirpated tumor.

**Conclusions:**

Two cases of pelvic SFT were diagnosed by immunohistochemical examination, RT-PCR and sequencing and successfully resected by laparoscopic surgery.

## Highlights

• The preoperative diagnosis of the solitary fibrous tumors is difficult irrespective of usage of the various imaging modalities.
• The technique of lateral pelvic lymph node dissection is useful for the extirpation of SFTs around the obturator cavity.
• Immunohistochemical examination of the specimens revealed STAT6 (+) and the NAB2-STAT6 fusion gene was detected in one case.

## 1 Introduction

Solitary fibrous tumors (SFT) [1] are mesenchymal tumors that can develop throughout the body. Complete excision is recommended for preventing local recurrence and metastasis. However, *en*-bloc R0 resection is not always easy due to the location and size of the tumors. A total of 43 cases of pelvic SFT were reported in the literature in Japan. However, in only 5 cases reported STAT6 protein overexpression by immunohistochemical staining, the product of the NAB2-STAT6 fusion gene, a driver mutation of the SFT [2]. Here, we report two cases of laparoscopic resection of SFT that were found accidentally in the deep pelvic cavity and review the reported cases in Japanese literatures. This manuscript has been reported in line with the SCARE criteria [3].

## 2 Presentation of cases

**Fig. 1:**
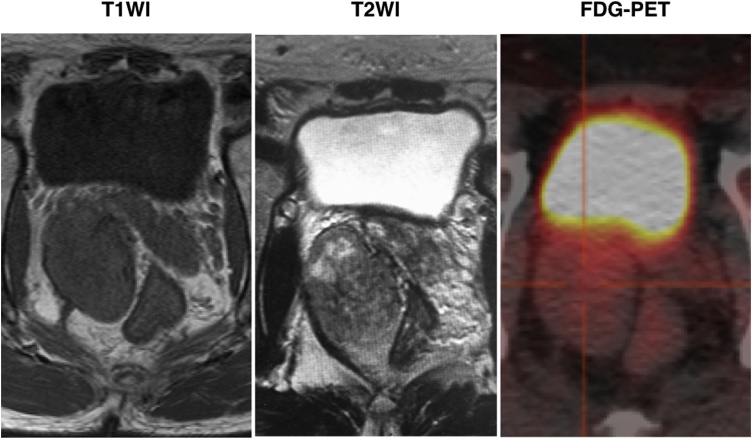
Preoperative MRI and FDG-PET findings of Case 1. Preoperative MRI showed a well-demarcated T1 low, T2 mixed intensity extramural tumor behind the seminal vesicle that was located between the right lateral wall of the low rectum and the right lateral pelvic wall. In FDG-PET, an accumulation of SUV was not shown corresponding to a 5.3 × 3.6 cm mass was identified.

Case 1: A 54-year-old male underwent a colonoscopy for the examination of colon polyps that did not detect any tumor lesions but revealed an extramural compression in the right anterior to lateral wall of the lower rectum. A CT scan revealed a well-demarcated encapsulated tumor (maximum length 60 mm) with contrast enhancement at the right anterior to lateral side of the rectum. A preoperative MRI showed a well-demarcated T1 low, T2 mixed intensity extramural tumor behind the seminal vesicle located between the right lateral wall of the low rectum and the right lateral pelvic wall (Fig. 1).

### 2.1 Surgical procedure (Fig. 2, video 1)

The preoperative diagnosis was gastrointestinal stromal tumor (GIST). Laparoscopic tumor resection was performed in March 2017. Based on the MRI findings, the peritoneum was incised outside the vesicohypogastric fascia to expose the internal obturator muscle following dissection between the left pelvic fascia and mesorectal fascia. Secondly, the tumor in the vesicohypogastric fascia was isolated from both sides. Finally, the tumor was removed from the vesicohypogastric fascia and levator ani muscles. The surgery lasted 388 min and the total amount of blood lost was 502 mL.

**Fig. 2:**
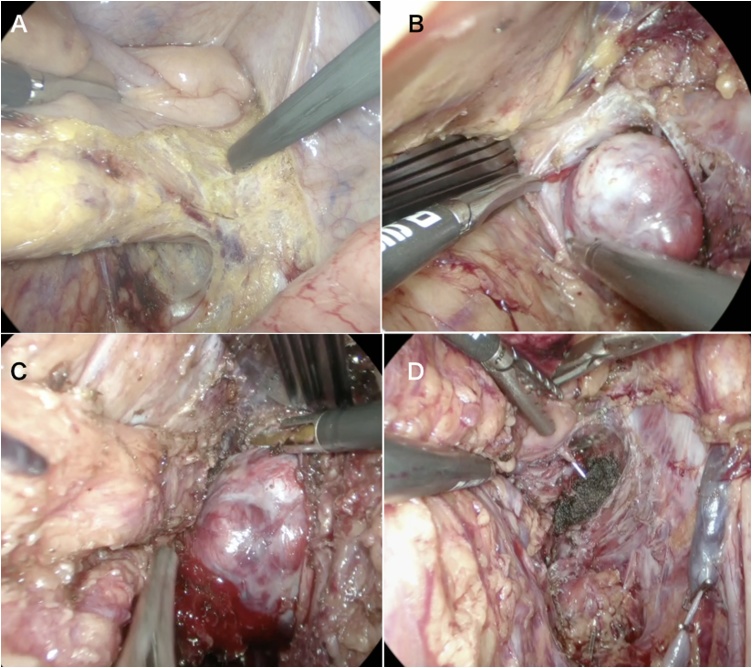
surgical procedure of case 1. A. Exposing medial side of the right pelvic plexus using total mesorectal excision dissection plane. B. Exposing lateral surface of the tumor following incision of the endopelvic fascia. C. Exposing medial surface of the tumor. D. Lateral pelvic side wall after tumor extirpation.

### 2.2 Pathological findings

Macroscopic findings revealed a smoothly lobulated mass (53 × 36 mm) encapsulated by a thin, translucent membrane. In addition, the cut surface appeared grey white to tan with a whorled pattern. Microscopic findings revealed the tumor consisted of spindle cell tumors complicated in bundles, and necrotic tissue with vascular and collagen fiber growth. Except for the hemostatic cauterization of the tumor vessels, the capsule structure was pathologically intact.

**Fig. 3:**
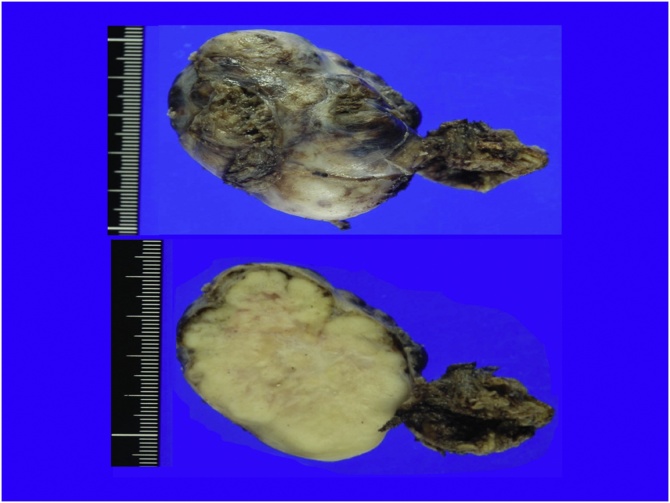
Macroscopic pathological findings of case 1 (cut surface).

**Fig. 4:**
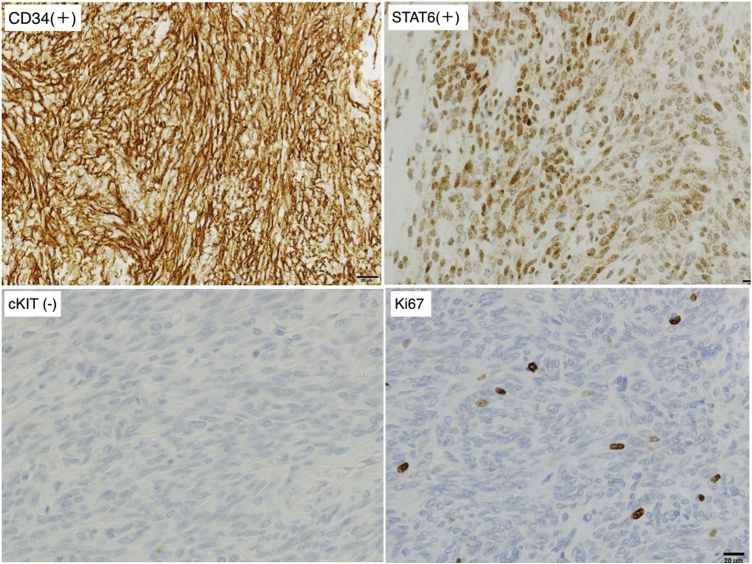
Immunohistochemical staining of case 1: CD34 (+), STAT6 (+), c-kit (−), Ki67.

The tumor was STAT6 (+), CD34 (+), DOG1 (−), cKIT (−), S100 focal (+), and Desmin (−) by immunohistochemistry. The NAB2-STAT6 fusion gene was also confirmed. Ki67 positive cells represented 7–8% and Fission image 4–5/50 HPF resulting in a diagnosis of malignant SFT (Fig. 3, Fig. 4). Using RT-PCR and sequencing, we detected the NAB2-STAT6 fusion gene, but a locus of genomic inversion was not detected. There was no recurrence 36 months after surgery by the CT scan.

**Fig. 5:**
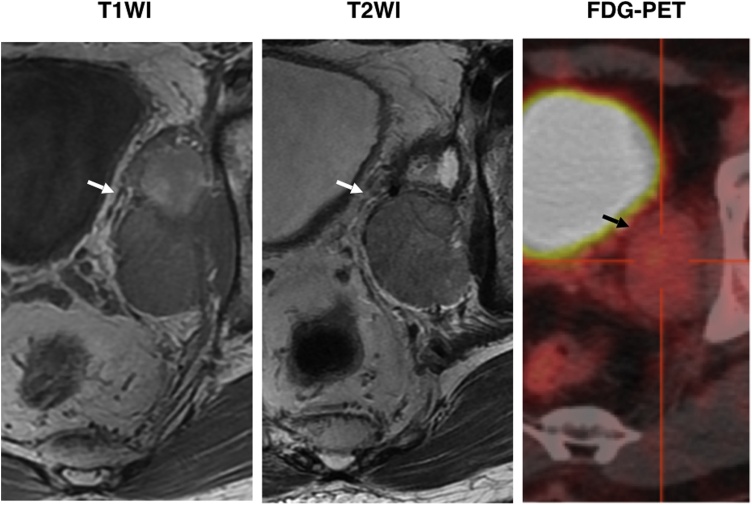
Preoperative MRI and FDG-PET findings of Case 2. Preoperative MRI after intraoperative biopsy revealed a well-demarcated, T1 and T2 low intensity tumor. In FDG-PET, an accumulation of SUV max 2.3 (im259) corresponding to a maximum length 40 mm mass was identified.

Case 2: A 43-year-old male was referred to our hospital complaining of pain in the right lower abdominal cavity and was diagnosed with appendicitis. A preoperative plain CT scan revealed a well-demarcated tumor (maximum length 40 mm) at the left obtulator fossa in contact with the left lateral wall of the rectum. He underwent laparoscopic appendectomy and a tumor biopsy via the retroperitoneal route. Histopathological examination of the biopsy specimen revealed it consisted of spindle tumor cells STAT6 (+), CD34 (+), DOG1 (−), cKIT (−), S100 (−), Desmin (−), CDK4 (−), PAX8 (−), and Ki67 positivity < 1% by immunohistochemistry. The tumor was diagnosed as SFT. A preoperative MRI after intraoperative biopsy revealed the tumor was a well-demarcated T1 and T2 low intensity tumor. In FDG-PET, an accumulation of SUVmax 2.3 corresponding to a mass was observed (Fig. 5).

### 2.3 Surgical procedure (Fig. 6, video2)

Based on the MRI findings, the Retzius cavity was dissected followed by dissection between the left side mesorectal fascia and pelvic plexus. The peripheral side of the neurovascular bundle was divided at the left lateral side of the prostate. The peritoneum was incised outside the left vesicohypogastric fascia to expose the internal obturator muscle. The tumor in the vesicohypogastric fascia was then isolated from both sides. Finally, the proximal side of the branches of the internal iliac vessels and the pelvic plexus were divided and the tumor removed together with the vesicohypogastric fascia. The surgery lasted 228 min and the total amount of blood loss was 168 mL.

**Fig. 6:**
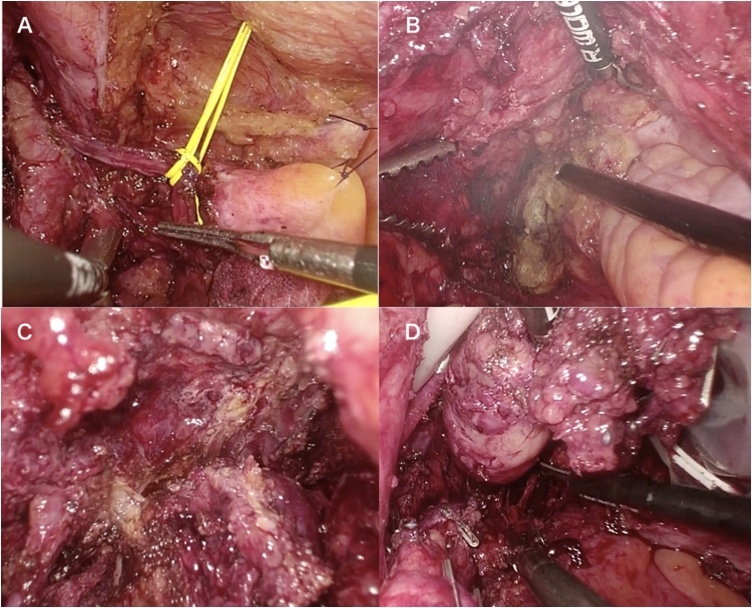
surgical procedure of case 2. A. Bladder and left VAS differences encircled and lifted up with yellow elastic tape. B. Dissection between the left pelvic plexus and mesorectal fascia. C. Dissection between the tumor lateral side and left obturator muscle. D. Tumor extirpation together with vesicohypogastric fascia.

### 2.4 Pathological findings

**Fig. 7:**
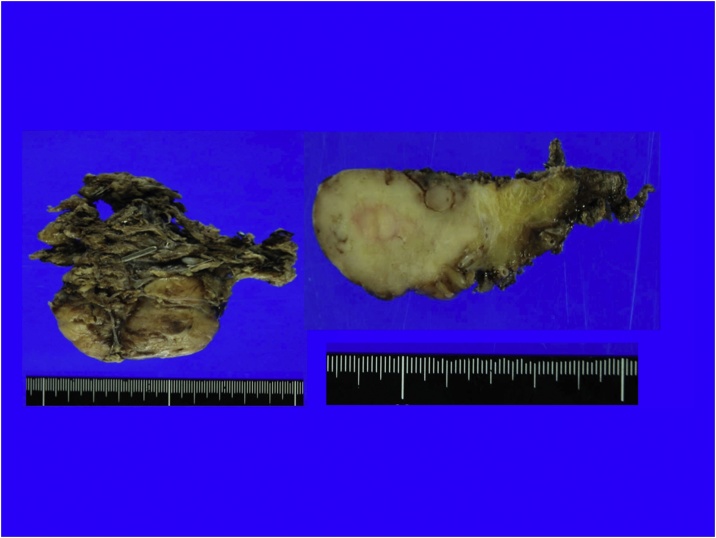
Macroscopic pathological findings of case 2 (cut surface).

**Fig. 8:**
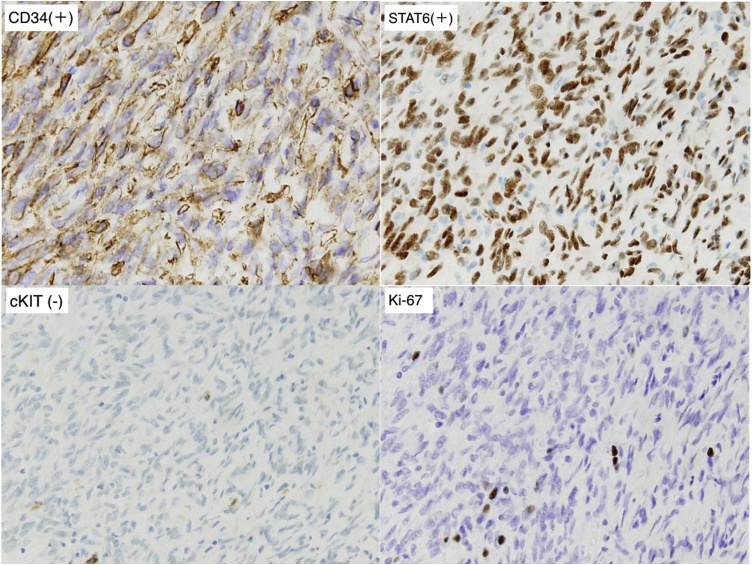
Immunohistochemical staining of case 2; CD34 (+), STAT6 (+), c-kit (−), Ki67.

Macroscopic findings revealed a smoothly lobulated mass (maximum length 40 mm) encapsulated by a thin membrane that was not detected by microscopic examination. The cut surface was grey white to tan with a whorled pattern. Microscopic examination revealed spindle tumor cells. The immunohistochemical findings were the same as the results of the biopsy sample (Fig. 7, Fig. 8). In this case, the tumor surface was macroscopically shiny smooth plane but pathological surgical margins were difficult to evaluated because tumor capsule was not existed. We could not detect the NAB2-STAT6 fusion gene by RT-PCR and sequencing. There was no recurrence 9 months after surgery as evaluated by the CT scan.

## 3 Discussion

SFTare rare neoplasias of mesenchymal origin with low grade malignant potential, deriving from a wide range of anatomic sites. Gold et al. reported that 16% of their series of SFT derived from the pelvic cavity [4]. Though pelvic SFT are relatively rare tumors, gastrointestinal surgeons, urologists and gynecologists have reported SFT in the pelvic cavity [5]. In this report we reviewed the manuscripts of pelvic SFT reported previously in Japan to understand the surgical outcomes (Table 1).

**Table 1:** Review of the pelvic SFT cases reported in Japanese literatures.

	n					
total number of patients	45			including current 2 cases		
number of manuscripts	41					
department of the institute of the first author	45			S 27, U 10, R 3, P 2, IM 1, G 1, E 1		
age (yo)	45	median (range)		58 (19–83)		
gender	45			M 29, F 16		
tumor location	45			pelvic cavity 19, retroperitoneal 11 obturator fossa 5, bladder 2 prostate 5, sigmoid colon-rectum 3		
symptoms	45			abdominal fullness 10, dysuria 4 pollakisuria 3, hypoglycemia 4 other symptoms 10, no symptom 14		
maximum length (cm)	44	median (range)		10 (0.85–29)		
MRI	35	T1	H	T2	H	4
			I		Mix	1
			Mix		Mix	2
			L		H (9), I (1), Mix (8), L (3)	21
			NA		I (1), L (1), Mix (5)	7

PET SUVmax	6	median (range)		2.2 (1.4–7.6)		
preoperative biopsy	17			SFT diagnosed by the biopsy 15		
operation	43			open 32, transsacal 5, lap 4 extraperitoneal 1, TUR-Bt 1		
mitotic index/10HPF	25	median (range)		1 (0–30)		
immunohistochemical	45	CD34		(+) 45		
	7	SFT		(+) 6, f/g (+) 1		
distant metastasis	43			(+) 3, (−) 40		
prognosis	38			alive 33, died 5		

n: “n” in each list indicates the number described in the manuscript.

P: pathology, U: urology, IM: internal medicine, S: surgery, E: emergency and critical care medicine, R: radiology, G: gynecology. MI: mitotic index. immunohistochemical: immunohistochemical staining of the specimen. MRI H/I/L/Mix; high/iso/low/mixed intensity. NA: not applicable. TUR-Bt: Transurethral resection of bladder tumor. f/g (+): NAB2-STAT6 fusion gene was detected but immunohistochemical staining of STAT6 was not described review of the pelvic SFT cases reported in Japanese literatures.

To the best of our knowledge, including our own cases, 45 cases of pelvic SFT have been reported in Japan of which 27 derived from the surgical department followed by 10 reports from urology, 3 from radiology and one from gynecology. There were no gender differences in the incidence with a median age of 58 (19–83) years. The median size of the tumor was 10 cm (0.85–29.0). Most of the cases were reported as large tumors with compression symptoms such as abdominal distension, frequent urination, and dysuria. Surgery was the only curative treatment, sometimes requiring a trans-sacral approach in addition to the open abdominal approach [[6], [7], [8], [9]]. In a report outside Japan, the sacrococcygeal bone was removed [10]. Arterial embolization was occasionally employed to prevent intra operative bleeding [11]. In these cases, the tumor occupied the pelvic space and the origin was hardly detected. In our cases, the size of the tumors, which were detected accidentally, were relatively small without any symptoms. Pelvic SFT were rarely located in the obturator fossa, except for one case. In this case, leg palsy was reported [12]. Four patients had hypoglycemia with a median tumor size larger than 10 cm (median: 14.3; range: 13.5–22) [[13], [14], [15], [16]].

SFT are not easy to diagnose preoperatively using imaging. Although MRI was often reported as T1 low and T2 high intensity, it was not always a characteristic finding of SFT. In FDG-PET, some reports revealed no uptake in the tumor. Case 2 showed a slight accumulation, but its significance is unknown. As a consequence, 17 cases were diagnosed by preoperative biopsy. In case 2, STAT6 immunohistochemical staining of the biopsy sample revealed the SFT. Most cases of SFTs behave as benign tumors, but 15–20% of them experience distant metastasis as well as local progression. Although *en*-bloc R0 resection is necessary also in neurosurgery, the rate of R0 resection varies depending on the tumor location [17].

In the cases presented here, tumors were detected below the peritoneal reflection involving the pelvic plexus and vesicohypogastric fascia with a relatively small size. As the resection of the pelvic plexus and vesicohypogastric fascia in combination with the tumor required clear anatomical recognition in the deep pelvic cavity throughout the operation, laparoscopic surgery was suitable for *en*-bloc resection of the tumors without injury. For the tumor located in the obturator fossa, the technique of laparoscopic lateral pelvic lymph node dissection that was widely accepted in high volume centers of colorectal surgery in Japan was available.

Recent studies revealed that the NAB2-STAT6 fusion gene was a driver mutation of SFT. The expression of NAB2-STAT6 fusion proteins had the early growth response (EGR)-binding domain of NAB2 fused to the activation domain of STAT6. Based on this review, only 5 cases were examined by STAT6 immunostaining (only one report described the presence of NAB2-STAT6 fusion gene) [13,14,[18], [19], [20]]. Based on the WHO classification, SFT have poor prognosis when they display a high cell density, strong nuclear abnormalities, and 5 or more fission and necrosis images in 10 high magnification fields [21,22]. However, this did not always correlate with the observed patient prognosis. Recently, prognosis is based on the type of fusion gene and the NAB2 exon 6–STAT6 exon 17/18 fusion gene [23]. Case 1 was classified as a malignant SFT by the WHO classification. However, there was no recurrence 29 months post-surgery. Case 2 was classified as benign based on the WHO classification, but caution should be taken depending on the exon site of the fusion gene.

## 4 Conclusions

In addition to this report, there are only 7 cases of pelvic SFT showing STAT6 immunohistochemical staining. It is expected that accumulation of data on NAB2-STAT6 fusion gene will elucidate the pathophysiology of pelvic SFT.

## Declaration of Competing Interest

No supportive foundations. No conflict interest has been declared by Yuki Matsui, Madoka Hamada, Fusao Sumiyama, Toshinori Kobayashi,Yuki Matsumi, Hisanori Miki, Mitsuaki Ishida, Hiroaki Kurokawa, Mitsugu Sekimoto, Yoko Sekita-Hatakeyama, Kinta Hatakeyama, Chiho Ohbayashi.

## Funding

No supportive foundations. No conflict interest has been declared by Yuki Matsui, Madoka Hamada, Fusao Sumiyama, Toshinori Kobayashi,Yuki Matsumi, Hisanori Miki, Mitsuaki Ishida, Hiroaki Kurokawa, Mitsugu Sekimoto, Yoko Sekita-Hatakeyama, Kinta Hatakeyama, Chiho Ohbayashi.

## Ethics approval

This study was approved by the Hospital Ethics Committee of Kansai Medical University (reference number #2019230: http://www.kmu.ac.jp/hirakata/hospital/2671t8000001356c.html).

## Consents

The patients’ written consent form for the published photos were obtained. In addition, written consent for the use of information for research and paper activities were obtained from all registered patients.

## Author contributions

Conception and design:YM, MH. Acquisition of the data: YM, MH, FS, TK, UM, HM.MI, HK,MS, YSH, KH, CO. Interpretation of the data: MH. Data analysis: MH. Drafting and revising the article: MH. Final approval: MH. All authors have read and approved the manuscript.

## Registration of research studies

Two cases of primary solitary fibrous tumor in the pelvis resected using laparoscopic surgery.

This study was approved by the Hospital Ethics Committee of Kansai Medical University (reference number #2019230: http://www.kmu.ac.jp/hirakata/hospital/2671t8000001356c.html).

## Guarantor

All authors were involved in preparation of this manuscript.

## Provenance and peer review

Not commissioned, externally peer-reviewed journal.
